# Discriminating flue-cured tobacco cultivars based on sensory quality and their underlying volatile and chemical profiles

**DOI:** 10.3389/fpls.2026.1802390

**Published:** 2026-04-15

**Authors:** Yanguo Ke, Shimeng Yang, Fang Zhao, Lihua Zheng, Zhongjian Fang, Ying Zou, Farhat Abbas, Yiwei Zhou, Junzhang Yang

**Affiliations:** 1 Yunnan Urban Agricultural Engineering and Technological Research Center, College of Economics and Management, Kunming University, Kunming, China; 2 Yunnan Tobacco company Zhaotong Company, Zhaotong, Yunnan, China; 3 Guangdong Provincial Key Laboratory of Ornamental Plant Germplasm Innovation and Utilization, Environmental Horticulture Research Institute, Guangdong Academy of Agricultural Sciences, Guangzhou, China; 4 Institute of Tropical Fruit Trees, Hainan Academy of Agricultural Sciences/Key Laboratory of Genetic Resources and Utilization of Tropical Fruits and Vegetables (Co-construction by Ministry and Province)/Key Laboratory of Tropical Fruit Tree Biology of Hainan Province, Haikou, Hainan, China

**Keywords:** biochemical composition, chemical-sensory correlation, cultivar selection, sensory attributes, tobacco, volatile organic compounds

## Abstract

Tobacco is a globally significant agricultural commodity, in which analytical chemistry plays a pivotal role for quality assessment. This study aimed to develop an integrated analytical strategy to decipher the quality traits of flue-cured tobacco cultivars. An integrated framework combining sensory evaluation, biochemical characterization, and volatile organic compound (VOC) profiling was applied to eleven flue-cured tobacco cultivars. Statistical analyses included hierarchical cluster analysis (HCA) and partial least squares-discriminant analysis (PLS-DA) based on VOC data, and Spearman correlation analysis (with Bonferroni correction) to explore relationships between sensory attributes, biochemical components, and VOCs. Sensory analysis categorized the cultivars into two groups: Group I (YN228, YN105, YN223, GY20, and YN87) scored higher in aftertaste, offensive taste, and moistness, while Group II (YN222, XY7, NC103, TZ113, GY2, and XZ01) excelled in aroma quality and cleanness. Biochemical profiling also revealed two distinct groups: Group I (YN228, TZ113, NC103, and YN87) had higher nitrogen and alkaloid contents, whereas Group II (YN222, XY7, GY20, XZ01, GY2, YN105, and YN223) exhibited elevated sugar and potassium levels. VOC-based HCA and PLS-DA identified three chemical clades and highlighted 75 VOCs (VIP > 1.0) as key differentiators. Significant correlations were established between biochemical components and sensory attributes; notably, total sugar content was positively correlated with irritancy, aroma quality, and smoke concentration. Specific VOCs, such as (furan-2-yl)methanol and 3-oxo-α-ionol, showed significant positive correlations with moistness and smoke concentration, respectively, while phenol was negatively correlated with aroma quality. This study establishes a reproducible sensory-omics framework that provides a robust analytical foundation for the quality assessment of agricultural products. The findings demonstrate the practical value of integrated analytical approaches in addressing complex quality assessment challenges, offering actionable insights for the evaluation and potential improvement of tobacco cultivars.

## 1 Introduction

Tobacco (*Nicotiana tabacum* L.) is a globally significant crop, with flue-curedtobacco being one of the most widely cultivated varieties due to its desirable sensory attributes and chemical composition (Jia et al., 2022). The quality of flue-cured tobacco is a crucial factor that influences its commercial value, shapes consumer preferences, and determines its various applications in the market. Traditionally, the evaluation of tobacco quality has relied on sensory assessments performed by expert panels. While this method has its advantages, it can be subjective and inconsistent (Chang et al., 2009). Extant research has extensively covered areas such as cultivation practices (Ding et al., 2024; Hu et al., 2021; Li et al., 2024b; Nghiem et al., 2024; Tang et al., 2023; Yao et al., 2024; Yi et al., 2025), broad chemical profiling (Fan et al., 2022; Fan et al., 2023; Jiang et al., 2025; Tie et al., 2024; Tsaballa et al., 2020; Zhu et al., 2025), quality grading (Chen et al., 2023; Wei et al., 2024; Xiang et al., 2020), and processing techniques for quality enhancement (Hu et al., 2023; Li et al., 2024a; Wu et al., 2021). Several studies have examined the relationship between the sensory quality of flue-cured tobacco and its aromatic volatile organic compounds (VOCs) or biochemical markers. For example, Liu et al. (2016) conducted a correlation analysis between sensory quality and key aroma components of red sun-cured tobacco from eight Chinese regions, identifying 22 aroma compounds closely associated with sensory smoking quality. Similarly, Shen et al. (2021) investigated the relationship between polyphenol content in tobacco leaves and sensory quality, reporting a significant positive correlation between polyphenols and attributes such as aroma quality, offensive taste, and aftertaste. Nevertheless, there remains limited reporting on the sensory quality differences among varieties of flue-cured tobacco and the key chemical constituents underlying these variations, which hinders the scientific evaluation of sensory quality and the selection of desirable varieties. This gap hinders the objective, scientific evaluation of sensory quality and data-driven cultivar selection.

Sensory quality is paramount for industrial competitiveness, as it directly reflects consumer acceptance (Krüsemann et al., 2019). This quality is fundamentally governed by the content, composition, and interactions of chemical constituents (Geng et al., 2025), which influence aroma, flavor, irritation, and aftertaste (Shun et al., 2011). While routine chemical analyses measure objective indicators like nicotine, total sugars, and chloride (Andrade et al., 2018), these metrics alone are insufficient. For instance, sugars balance nitrogenous compounds and mitigate irritation (Wang et al., 2016), and nicotine impacts physiological strength (Farrokh et al., 2012), yet they do not fully capture flavor complexity.

Flavor complexity is largely attributed to volatile organic compounds (VOCs). Over 400 VOCs, including aldehydes, ketones, alcohols, and terpenoids, have been identified in tobacco, each imparting unique sensory notes (e.g., sweet, floral, nutty) (Jiang et al., 2024). Moreover, volatile acids, which are released into smoke during consumption, significantly contribute to the overall flavor profile (Geng et al., 2023). Key aroma formation pathways, such as the Maillard reaction between amino acids and sugars during curing and aging, generate numerous flavorants (Wahlberg et al., 1977; Kanzler et al., 2017). Despite their recognized importance, VOC profiles—encompassing both neutral and acidic volatiles—are often studied in isolation rather than as an integrated system explaining perceptible sensory differences among cultivars.

Therefore, the present study hypothesizes that distinct flue-cured tobacco cultivars possess unique sensory signatures driven by characteristic volatile and chemical profiles, enabling their discrimination through multivariate statistics. To test this, we aimed to: (1) assess the sensory attributes of eleven cultivars via expert panel evaluation; (2) characterize their conventional chemical indicators and volatile compound profiles (encompassing a broad range of VOCs) using GC-MS; and (3) employ multivariate statistical models to identify the key discriminatory compounds and establish correlations between sensory quality and underlying chemical constituents. This integrated approach seeks to provide a more objective framework for quality evaluation, reduce reliance on subjective judgment, and offer insights for targeted breeding and processing optimization. Future research may expand this methodology to other cultivars and explore the genetic basis of these trait variations.

## 2 Materials and methods

### 2.1 Materials and flued treatment

Tobacco samples were cultivated and collected from the production areas of Yiliang County, Zhaotong City, Yunnan Province, China (27.63° N, 104.06° E). The samples included eleven cultivars named YN228, YN222, XZ01, NC-103, TZ113, GY20, XY7, GY2, YN87, YN105, and YN223. The middle portions of the leaves from these various cultivars were harvested and flued using a three-stage process: yellowing, color-fixing, and stem drying. This method gradually removes moisture while preserving the quality of the leaves. Three biological replicates were prepared for each cultivar. Plants were grown in red soil and cultivated in three independent test field regions under consistent conditions. From each region, the third leaf (counting from the top) was collected from five individual plants at the same developmental position. Leaves from the same cultivar across the three field regions were pooled to form one composite biological replicate. All samples were harvested in 2024.

During the yellowing stage (35-42°C, ≥80% RH), high humidity and moderate temperatures promote chlorophyll degradation and enhance enzymatic activity, resulting in softer leaves and the production of fragrance precursors. In the color-fixing stage (42-48°C, ~70% RH), the temperature is increased while humidity is decreased to maintain the yellow hue, prevent enzymatic browning, and initiate dehydration. Finally, the stem drying stage (48-68°C, ≤60% RH) employs higher temperatures and vigorous airflow to dry the midrib and veins, which helps prevent mold and ensures proper storage (Wang et al., 2016).

### 2.2 Sensory evaluation

Sensory evaluation was performed in collaboration with Yunnan Jiahui Testing Technology Co., Ltd., a professional testing company that maintains a specialized panel of trained assessors and has extensive experience in tobacco sensory analysis. The sensory quality of cigarettes was assessed according to the Chinese National Standard GB 5606.4—2005, GB/T 5606.1, and GB/T 16447, with minor modifications as detailed in the following procedure. Sensory evaluation was conducted by a panel of at least seven trained assessors in a controlled environment. All procedures-including sample preparation, moisture conditioning, assessor training, environment control, attribute definitions, and scoring protocols-were adhered to as mandated by the standard. To minimize bias, the presentation order of the tobacco samples was fully randomized for each assessor and each session. All samples were coded with three-digit random numbers, and assessors were blinded to sample identities throughout the evaluation process. Ten attributes were evaluated: aroma profile, aroma volume, aroma quality, smoke concentration, irritancy, smoke strength, offensive taste, cleanness, moistness, and after taste. Each attribute was individually scored on a 10-point scale based on the descriptive criteria provided in the standard, with minor adjustments made as needed. For each attribute, the average score across all panelists was calculated, rounded to 0.01, and overall quality was expressed as the mean of the six attributes, rounded to 0.1. Any sample exhibiting serious sensory defects was deemed non-conforming. The results were reported as the mean of two parallel evaluations.

### 2.3 Analysis of total sugars, reducing sugars, total alkaloids, chloride, potassium, and nitrogen content

Total sugars were determined using the anthrone–sulfuric acid spectrophotometric method. Approximately 0.2 g of dried, homogenized tobacco powder was extracted with 20 mL of deionized water at 80°C for 30 min. After centrifugation and filtration, 1.0 mL of the extract (diluted as needed) was reacted with 5.0 mL of ice-cold anthrone reagent. The mixture was heated in a boiling water bath for 10 min, rapidly cooled, and absorbance was measured at 620 nm. Quantification was performed using a glucose calibration curve (0–100 µg/mL). Results are expressed on a dry-weight basis.

Reducing sugars were determined following industry standard YC/T 159-2019. Approximately 0.25 g of sample was extracted with 25 mL of 5% acetic acid by shaking at >150 rpm for 30 min. After filtration, the extract was analyzed by continuous-flow analysis. Reducing sugars reacted with p-hydroxybenzoic acid hydrazide (PAHBAH) in an alkaline medium at 85°C to form a yellow azo compound, with absorbance measured at 410 nm. Calibration was performed with glucose standards prepared in 5% acetic acid. Results are expressed as glucose equivalents.

Total alkaloids (expressed as nicotine equivalents) were analyzed according to YC/T 468-2021. A 0.25 g sample was extracted with 25 mL of extractant (deionized water or 5% acetic acid) by shaking for 30 min at room temperature. After filtration, alkaloids in the extract were reacted sequentially with p-aminobenzenesulfonic acid and an in-situ-generated oxidant (from potassium thiocyanate and dichloroisocyanurate) to yield a colored complex, with absorbance measured at 460 nm. A nicotine calibration curve was used for quantification.

Chloride was determined according to YC/T 162–2011 using a continuous-flow-analysis spectrophotometric method. Approximately 0.25 g of homogenized tobacco was extracted with 25 mL of deionized water by shaking at >150 rpm for 30 min. The filtrate was analyzed on a continuous-flow analyzer equipped with dialysis and colorimetric detection. Chloride ions reacted with mercuric thiocyanate to release thiocyanate, which forms a red ferric thiocyanate complex, detected at 460 nm. Calibration was performed with sodium chloride standard solutions, and extracts were diluted as required. Results are expressed on a dry-weight basis.

Nitrogen was quantified following YC/T 161-2002. Approximately 0.1 g of powdered sample was digested with 0.1 g HgO, 1.0 g CuSO_4_, and concentrated H_2_SO_4_ in a block digester (150°C for 1 h, then 370°C for 1 h until the solution clarified). After cooling and dilution, nitrogen in the digest reacted with alkaline potassium sodium tartrate, potassium ferricyanide, and sodium hypochlorite to form a blue complex, with absorbance measured at 660 nm. Calibration was based on ammonium sulfate standards.

Potassium was measured according to YC/T 217-2007. A 0.25 g sample was extracted with 25 mL of deionized water by shaking for 30 min. After filtration, potassium in the extract was determined by continuous-flow analysis coupled with flame photometry, calibrated with KCl standard solutions.

All analyses were performed on data derived from three biological replicates.

### 2.4 Determination of volatile organic compounds in flue-cured tobacco products from different cultivars

Headspace solid-phase microextraction (HS-SPME), a solvent-free and sensitive preconcentration technique, was employed to extract volatile organic compounds (VOCs) from flue-cured tobacco samples, followed by analysis with gas chromatography–mass spectrometry (GC–MS). Briefly, approximately 1.0 g of tobacco was placed into a 20 mL headspace vial, which was then sealed with a crimp cap. All samples were prepared and analyzed in three biological replicates. Extraction was performed using a SPME with a Triplus RSH autosampler (Thermo Fisher Scientific, USA) equipped with a DVB/Carbon WR/PDMS fiber (50/30 μm; Thermo Fisher Scientific). The samples were pre-incubated at 90°C with shaking at 250 rpm for 5 min, followed by headspace extraction for 20 min. The fiber was subsequently thermally desorbed in the GC injection port at 240°C for 3 min. The total run time per sample was 41 min. A procedural blank (empty vial put through the full SPME-GC-MS process) was analyzed. Any peaks present in the blank were subtracted from the sample data, and features with a signal-to-noise ratio (S/N) < 10 were filtered out to ensure data quality.

Chromatographic separation was performed on a Trace 1610 gas chromatograph coupled to a TSQ 9610 mass spectrometer (Thermo Fisher Scientific, USA) using a DB-Wax capillary column (30 m × 0.25 mm × 0.25 μm). Helium served as the carrier gas at a constant flow rate of 1.2 mL/min. Injection was performed in split mode (5:1) with an injection volume of 1 μL. The oven temperature program was as follows: initial temperature 50°C, increased to 100°C at 5°C/min, then to 150°C at 3°C/min, and finally to 240°C at 10°C/min, with a final hold of 2 min. The transfer line and ion source temperatures were set to 240°C and 280°C, respectively. The mass spectrometer was operated in electron ionization (EI) mode at 70 eV, scanning an m/z range of 40–400.

The Wiley 12th mass spectral database was employed for metabolite identification, matching molecular characteristic peaks to detect and identify metabolites in the biological system as comprehensively as possible, thereby maximizing coverage of the overall metabolome. Raw data were preprocessed using Chromeleon™ CDS software, which included peak extraction, baseline correction, deconvolution, peak integration, and peak alignment. Metabolites were tentatively identified based on mass spectrum matching and retention index comparison using the Wiley 12th database. Identified metabolites were subsequently annotated for function and classification using major databases, including KEGG, HMDB, and LIPID MAPS. For relative quantification, the XCMS package in R (v3.3.2) was used to perform deconvolution and peak integration of the detected spectra. To correct for potential minor variations in sample amount or injection, the raw peak area of each detected compound was normalized to the total peak area (TPA) of all detected compounds within the same sample. All analyses were performed on three biological replicates.

### 2.5 Statistical analysis

Unless otherwise specified, all analyses were conducted in the R programming environment (R Core Team, 2023). Hierarchical clustering analysis (HCA), principal component analysis (PCA), one-way analysis of variance (ANOVA), and correlation analysis (Pearson and Spearman) were performed using built-in functions in R. The significance of correlation coefficients was assessed using *p*-values adjusted via the Bonferroni correction method. The HCA heatmap was generated using the “ComplexHeatmap” package (Gu et al., 2016). Permutational multivariate analysis of variance (PERMANOVA) was performed using the “vegan” package. Partial least squares discriminant analysis (PLS-DA) and variable importance in projection (VIP) value calculations were carried out using the “MetaboAnalystR” package (Chong and Xia, 2016). The optimal number of latent components for the PLS-DA model was determined through 5-fold cross-validation, with selection based on model accuracy, goodness-of-fit (*R^2^*), and predictive performance (*Q^2^*). The significant association network was visualized using Cytoscape 3.10.4 (Shannon et al., 2003).

## 3 Results

### 3.1 Sensory evaluation and clustering analysis of different tobacco cultivars

**Figure 1:**
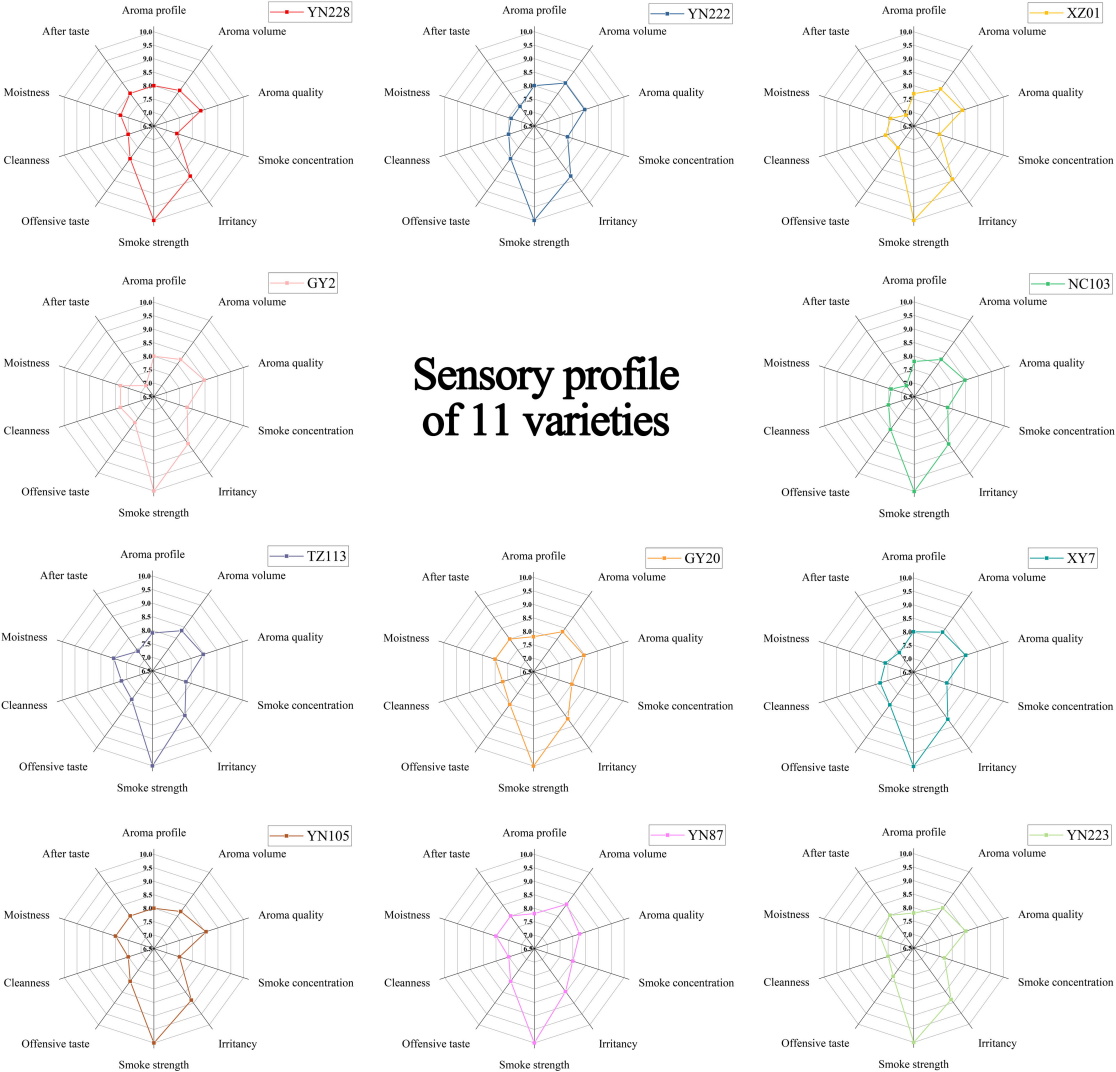
Sensory evaluation analysis of 11 flue-cured tobacco samples.

The sensory analysis reveals a high-quality, yet differentiable, phenotypic landscape among the eleven flue-cured tobacco cultivars (**Figure 1**). Aroma attributes displayed distinct and divergent performance patterns among cultivars. The aroma profile scores ranged from 7.7 to 8.0, with cultivars YN228, YN222, XY7, GY2, and YN105 achieving the maximum (8.0). For aroma quality, values varied between 8.27 and 8.53. Cultivars XY7, YN105, and YN223 formed the top tier with a score of 8.53, while YN228 (8.33) and YN87 (8.27) occupied the lower end of the spectrum. Notably, aroma volume presented the greatest relative spread, with YN87 (8.53) demonstrating superior intensity and YN228 (8.13) the lowest, positioning it as a key variable for sensory impact.

Sensory comfort and cleanness attributes provided critical discriminatory power. Irritancy emerged as a primary differentiator, with scores spanning a 0.46-point range (8.47 for YN87 to 8.93 for XZ01). Cultivars YN105 and YN223 also exhibited relatively high irritancy (8.87), whereas YN228 and YN222 scored 8.80. Offensive taste scores varied from 7.5 to 8.0, with a majority of cultivars, including YN228, GY20, and XY7, achieving the top score. Cleanness scores were closely clustered between 7.5 and 7.8, with XY7 and GY2 recording the highest value. Variation in other key sensory characteristics further defined cultivar-specific signatures. Smoke concentration scores ranged from 7.4 (YN228) to 8.0 (GY20, YN87), highlighting differences in perceived smoke density. High performance in moistness and aftertaste (scores of 8.0) was observed in five cultivars (TZ113, GY20, YN87, YN105, and YN223), suggesting a shared physiological or compositional basis for sustained mouthfeel and flavor persistence.

Synthesis of these profiles confirms that cultivar superiority is attribute-contingent, defined by unique trait combinations rather than uniform excellence. For instance, YN87 was characterized by the highest aroma volume, the lowest irritancy, and strengths in smoke concentration, moistness, and aftertaste. XY7 excelled in aroma profile, aroma quality, and offensive taste. GY20 demonstrated advantages in smoke concentration, moistness, and aftertaste. This detailed profiling establishes a foundation for selecting cultivars aligned with specific sensory design objectives.

**Figure 2:**
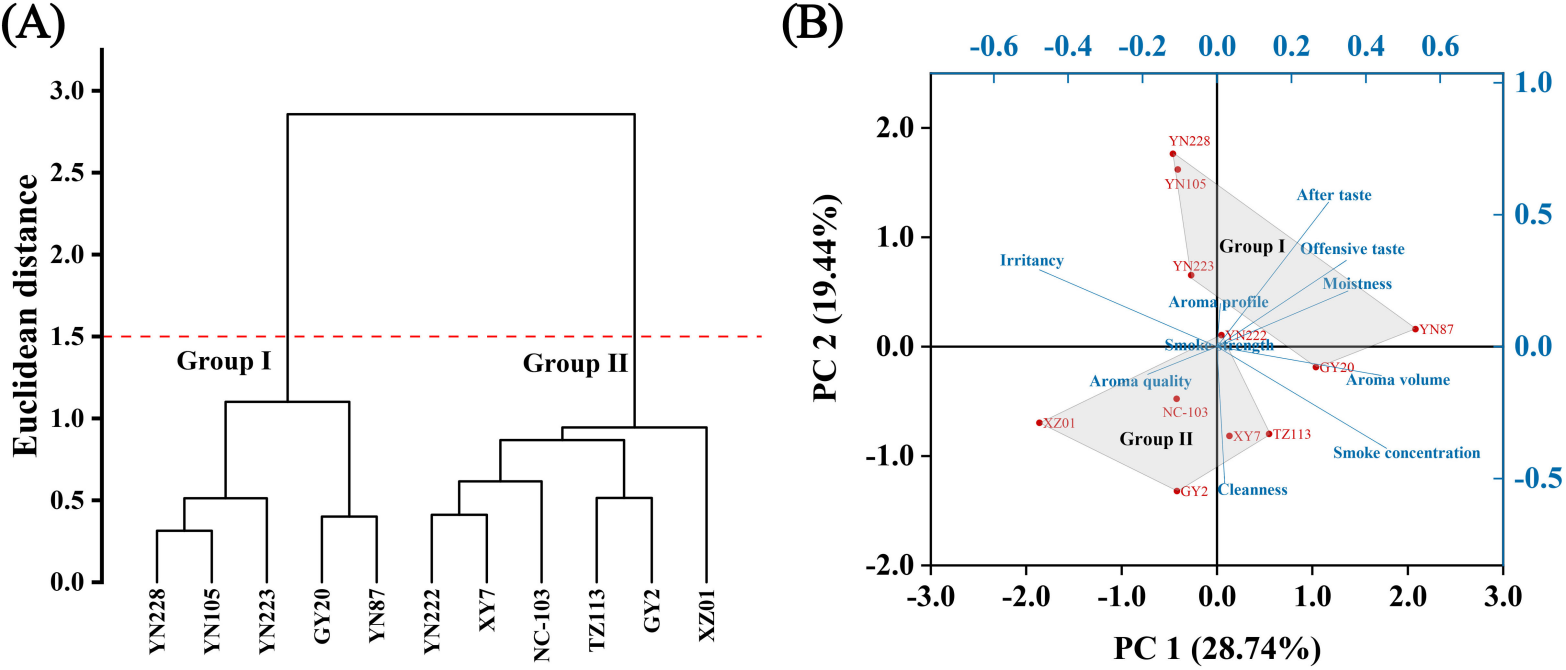
Diversity analysis of 11 flue-cured tobacco samples based on 10 sensory evaluation indices. **(A)** Hierarchical clustering analysis; **(B)** Biplot of principal component analysis (PCA).

Correlation analysis established foundational relationships between key sensory attributes. Significant positive correlations were identified between aroma volume and smoke concentration, and between moistness and aftertaste, suggesting these pairs of attributes may share underlying compositional or perceptual drivers. In contrast, a significant negative correlation was observed between smoke concentration and irritancy (**Supplementary Table 1**). HCA delineated the 11 varieties into two primary sensory groups Using Euclidean distance, the dendrogram revealed a distinct bifurcation: Group I comprises YN228, YN105, YN223, GY20, and YN87; Group II includes YN222, XY7, NC-103, TZ113, GY2, and XZ01 (**Figure 2A**). This classification offers valuable information regarding the relationship between varietal characteristics and sensory quality attributes.

PCA further elucidated the multidimensional sensory relationships and specific attribute contributions (**Figure 2B**). The first principal component (PC1, 28.74%) and the second principal component (PC2, 19.44%) account for a substantial proportion of the variance in the data (**Supplementary Table 2**). PCA score plot corroborated the clustering structure revealed by HCA. Samples in Group I predominantly clustered in the upper-right quadrant, characterized by higher scores for aroma profile, aftertaste, offensive taste, and moistness. Conversely, samples in Group II were located mainly in the lower-left quadrant and were associated with higher scores for aroma quality, cleanness, and smoke concentration (**Supplementary Table 3**). However, PERMANOVA result indicated no significant overall sensory differences among varieties (*F* = 0.674, *p* = 0.553), suggesting relatively limited variation in holistic sensory profiles across the groups.

### 3.2 Biochemical composition and diversity analysis of tobacco cultivars

**Figure 3:**
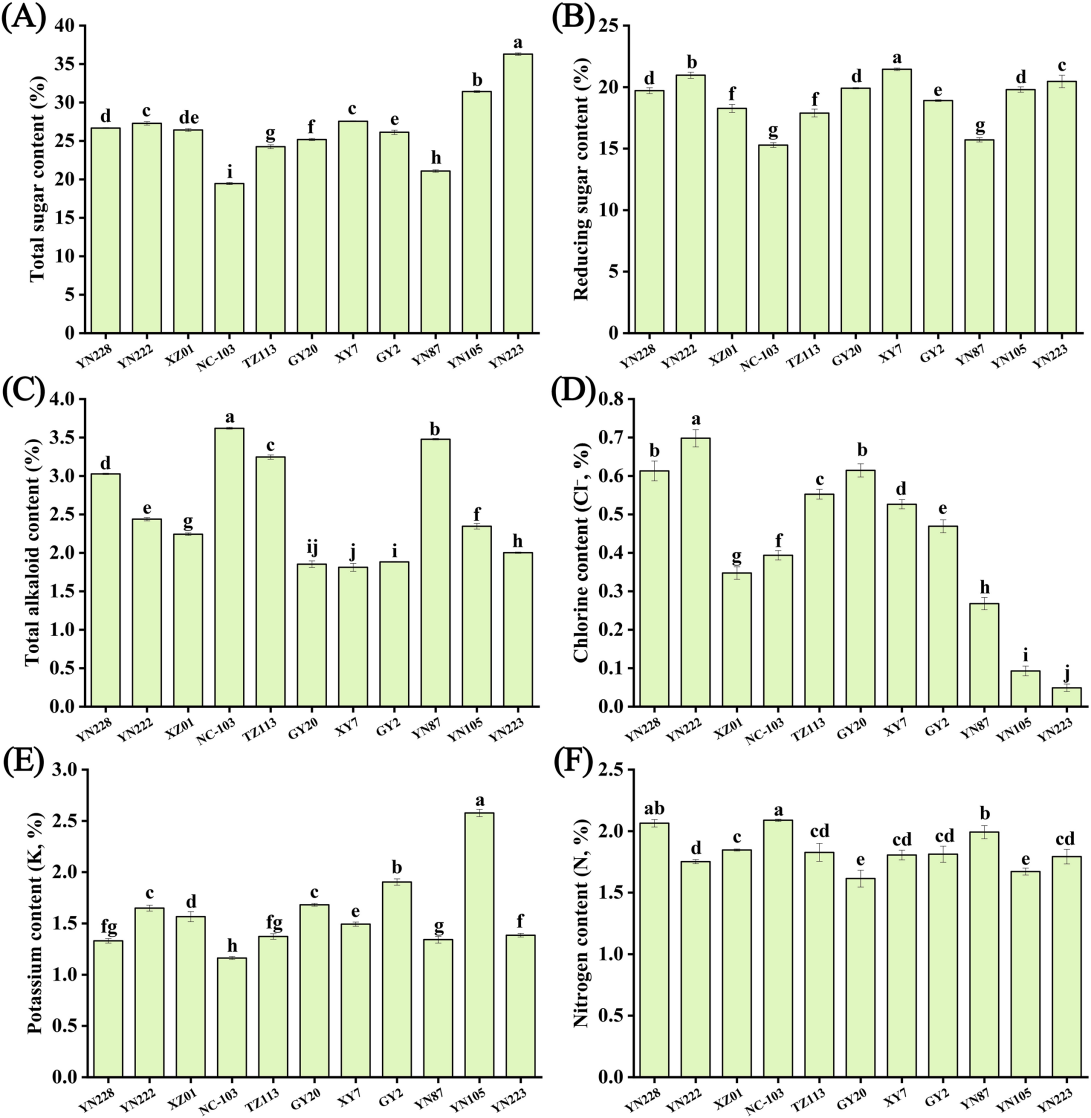
Comparative analysis of **(A)** total sugar content, **(B)** reducing sugar content, **(C)** total alkaloid content, **(D)** chlorine content, **(E)** potassium content, and **(F)** nitrogen content in 11 flue-cured tobacco samples. Different lowercase letters above the bars indicate statistically significant differences at a significance level of *p* < 0.05 according to one-way ANOVA.

The biochemical profiles of eleven flue-cured tobacco cultivars exhibited significant compositional diversity, establishing a quantifiable chemical basis for their sensory differentiation. Six key compound groups—total sugars, reducing sugars, total alkaloids, chlorine, potassium, and total nitrogen—were quantified, revealing distinct and non-overlapping compositional patterns among cultivars (**Figure 3**). Significant compositional diversity was observed among the cultivars, providing a chemical basis for their sensory variation. Analysis of sugar content revealed distinct profiles, with cultivar YN223 exhibiting the highest levels of total sugar (**Figure 3A**) and XY7 exhibiting the highest levels of reducing sugar (**Figure 3B**). In contrast, cultivars YN87 and NC103 showed relatively lower sugar contents. Cultivar YN105 also demonstrated elevated sugar levels, though lower than those of YN223. Other key chemical components further delineated complex cultivar-specific chemical signatures. For total alkaloids (**Figure 3C**), NC103 and YN87 had the highest concentrations, whereas XY7 was among the lowest. Chlorine content (**Figure 3D**) was highest in YN222 and lowest in YN223. Potassium levels (**Figure 3E**) were greatest in YN105, followed by GY2, and lowest in NC103. Total nitrogen content was higher in NC103, and YN228, and lower in YN105 and GY20 (**Figure 3F**). Synthesis of these profiles confirmed that no single cultivar exhibited a universally superior chemical composition; instead, each presented a unique biochemical configuration. For example, YN223 was characterized by high sugar content but the lowest chlorine level, whereas YN222 showed a combination of high sugar, high chlorine, and relatively lower nitrogen content. These distinct compositional profiles underlie the differential sensory potential of each cultivar, as explored in subsequent correlation analyses.

Correlation analysis revealed significant interrelationships among the key chemical components (**Supplementary Table 4**). Specifically, a strong positive correlation was observed between reducing sugar and total sugar content, and between total alkaloid and nitrogen content. Conversely, reducing sugar content exhibited significant negative correlations with both total alkaloid content and nitrogen content. Additionally, a significant negative correlation was observed between total alkaloid content and potassium content. Furthermore, potassium content and nitrogen content also demonstrated a significant negative correlation.

**Figure 4:**
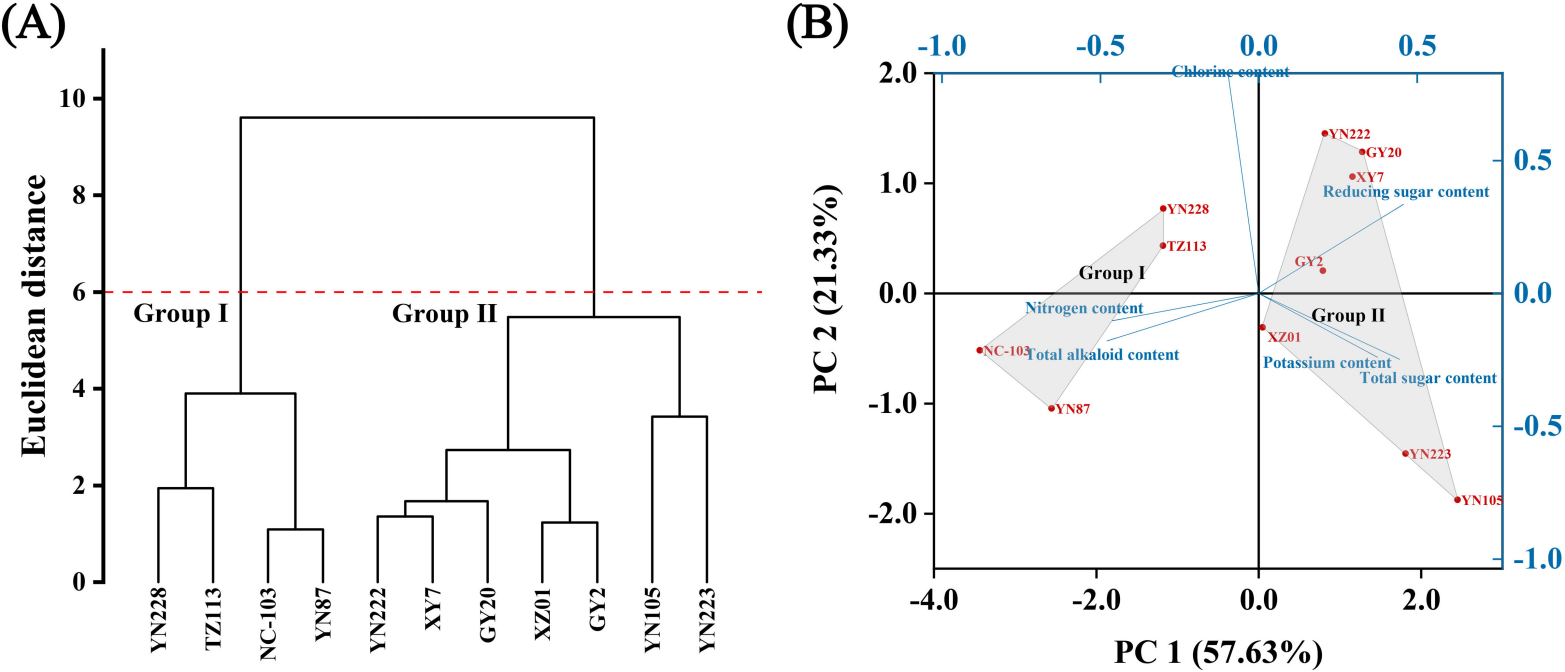
Diversity analysis of 11 flue-cured tobacco samples based on 6 physiological indices. **(A)** Hierarchical clustering analysis; **(B)** Biplot of principal component analysis (PCA).

Both HCA and PCA consistently delineated the 11 cultivars into two distinct chemotypic groups (**Figure 4**). HCA separated the cultivars into two primary clusters: Group I (YN228, TZ113, NC-103, and YN87) and Group II (YN222, XY7, GY20, XZ01, GY2, YN105, and YN223). This classification indicates greater intra-group similarity in chemical profiles and distinct inter-group divergence (**Figure 4A**). PCA further resolved the specific chemical drivers of this group separation (**Figure 4B**, **Supplementary Table 5**). The first two principal components (PC1: 57.63%; PC2: 21.33%) explained 78.96% of the total compositional variance. The loading plot (**Supplementary Table 6**) indicates that PC1 primarily represents a gradient from high total alkaloid and chlorine content (negative PC1) to high total sugar and reducing sugar content (positive PC1). Accordingly, Group I cultivars (YN228, TZ113, NC-103, YN87) are positioned on the negative PC1 axis, characterized by elevated alkaloid and chlorine levels. In contrast, Group II cultivars (YN222, XY7, GY20, XZ01, GY2, YN223, YN105) are distributed on the positive PC1 axis, associated with higher sugar concentrations.

Within these broad groups, individual cultivars exhibited distinct chemical signatures. For instance, within Group I, NC-103 and YN87 demonstrated particularly high levels of nitrogen and total alkaloids. Within Group II, YN223 and YN105 were notable for their elevated potassium and total sugar content, while YN222, GY20, and XY7 showed the highest reducing sugar levels. In contrast, YN228 and TZ113 (Group I) were distinguished by their high chlorine content. This intricate chemical diversity underscores the varied metabolic and physiological backgrounds of the cultivars, providing a material basis for their divergent sensory properties. PERMANOVA revealed significant between-group differences in physiological indices (*F* = 6.72; *p* = 0.028), indicating that the grouped physiological traits differ meaningfully and may constitute an important factor underlying the subtle variations observed in sensory characteristics.

### 3.3 Qualitative, quantitative, and diversity assessment of volatile organic compounds in eleven flue-cured tobacco cultivars

**Figure 5:**
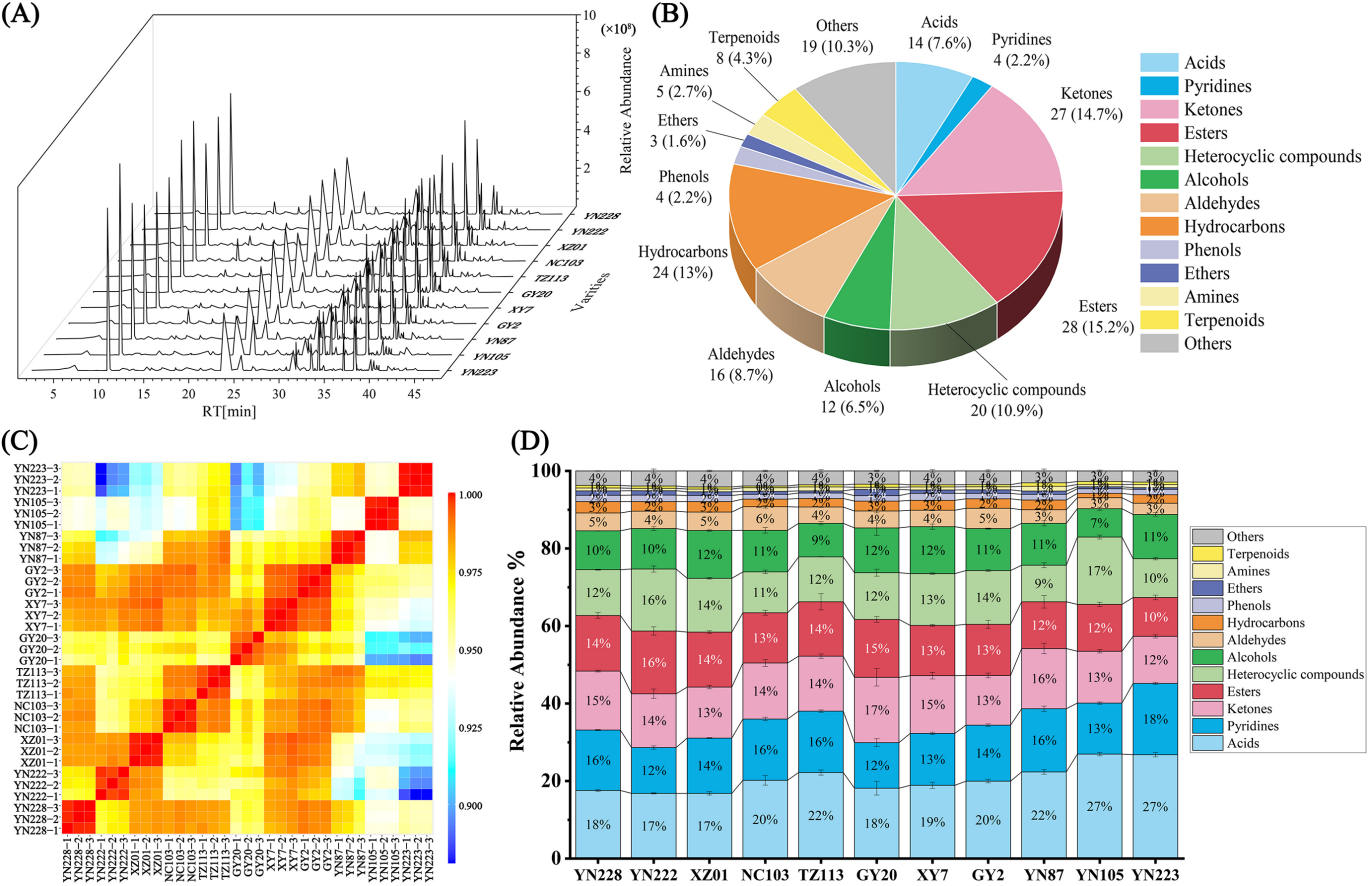
Qualitative and quantitative analysis of volatile organic compounds (VOCs) in 11 flue-cured tobacco samples detected by HS-SPME/GC-MS. **(A)** Total ion chromatogram (TIC); **(B)** Count and proportion of different VOC categories; **(C)** Heatmap of Pearson correlation coefficient matrix; **(D)** Comparative analysis of relative content of different VOC categories.

To delve deeper into the VOCs present in 11 flue-cured tobacco cultivars, HS-SPME-GC-MS analysis was employed. The representative total ion chromatograms (TICs) of the 11 cultivars (**Figure 5A**) display similar peak patterns across major compound categories, indicating stable GC-MS performance and a largely conserved VOC profile, while certain peak intensities demonstrate cultivar-specific variations. The distribution patterns of diverse chemical components across these eleven cultivars are depicted in **Figure 5**. A comprehensive suite of 184 VOCs was identified, encompassing the various chemical categories described below (**Supplementary Table 7**). Esters comprise 15.2% of the total composition, followed by ketones at 14.7% and hydrocarbons at 13% (**Figure 5B**). Additional significant categories comprise heterocyclic compounds (10.9%), aldehydes (8.7%), acids (7.6%), and alcohols (6.5%). Smaller proportions are observed in groupings such as pyridines (2.2%), amines (2.7%), and ethers (1.6%), whereas phenols and terpenoids account for 2.2% and 4.3%, respectively.

Pearson correlation analysis was performed to assess the stability of biological replicates. The results demonstrated that the correlation coefficients between replicates of the same cultivar were consistently higher than those observed between different cultivars, indicating good reproducibility within each cultivar (**Figure 5C**). The statistics indicate that the composition is significantly affected by acids, pyridines, esters, ketones, and heterocyclic coompounds, which are probable primary drivers of the aroma profiles of the cultivars (**Figure 5D**).

**Figure 6:**
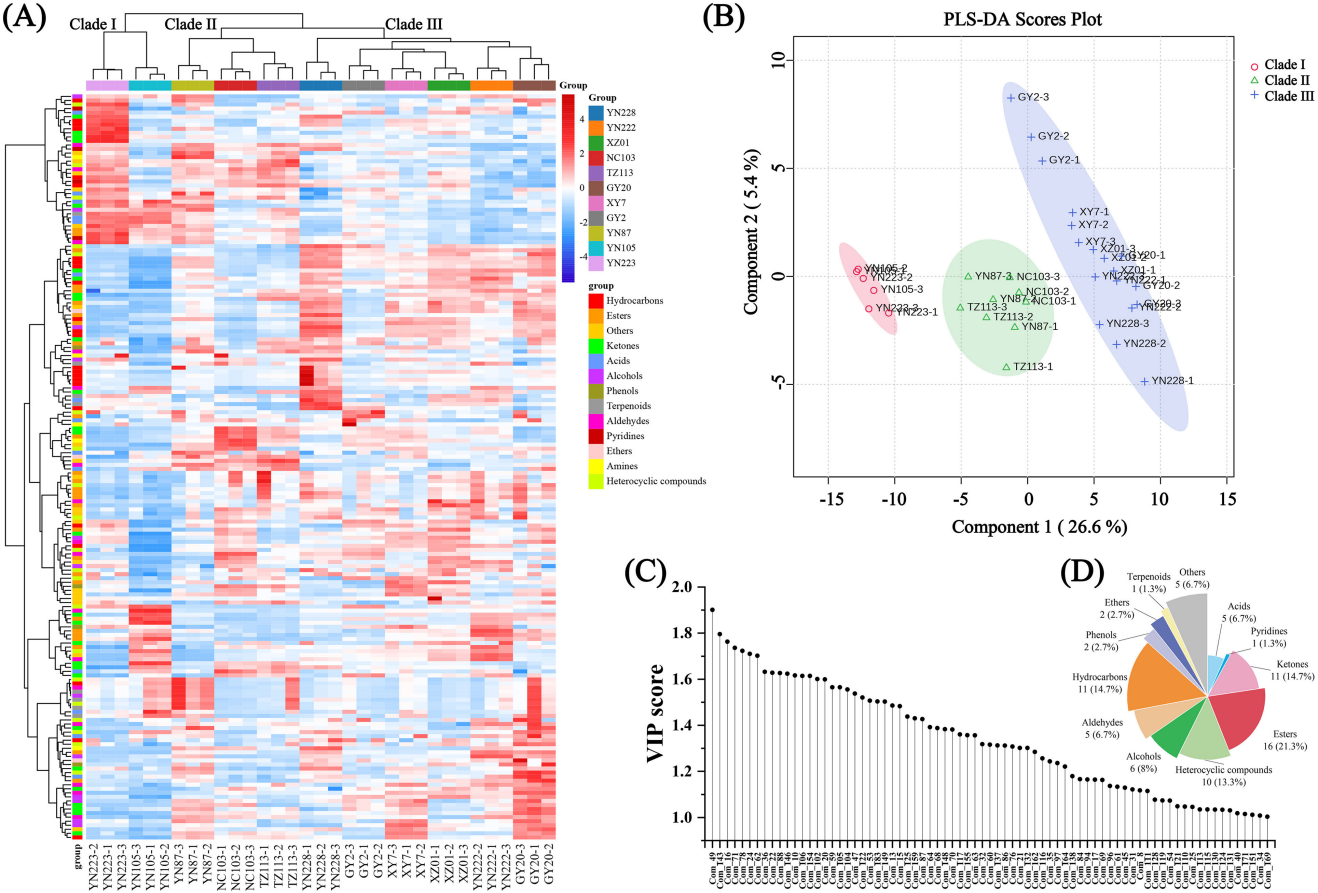
Diversity analysis of 11 flue-cured tobacco samples based on 184 VOCs. **(A)** Hierarchical clustering analysis; **(B)** Partial least squares-discriminant analysis (PLS-DA) score plot; **(C)** 75 key VOCs with variable importance in projection (VIP) > 1. **(D)** Classification and statistics of 75 key VOCs.

The HCA heatmap demonstrated that the three replicates of each cultivar consistently clustered together, indicating good experimental reproducibility (**Figure 6A**). As shown in the heatmap, the chemical compositions of the cultivars are divided into three distinct clades: Clade I, Clade II, and Clade III. YN223 and YN105 are grouped in Clade I, YN228, YN222, and GY20, into Clade II, while GY2, XY7, XZ01, YN87, NC103, and TZ113 are categorized into Clade III, exhibiting distinct chemical profiles. The heatmap indicates substantial quantitative variations in VOC emissions across clades, corroborating their chemical classification.

To further investigate clade-specific VOC signatures, performed PLS-DA was performed. **Figure 6B** displays the PLS-DA score plot, with the first two components accounting for 26.6% and 5.4% of the variation, respectively. Cross-validation results indicated that the optimal model was achieved with three components, yielding a *Q^2^* of 0.93966 and a *R^2^* of 0.99062, demonstrating both high predictive performance and model stability. The cultivars are categorized according to their chemical profiles, with Clade I denoted in red, Clade II in blue, and Clade III in green. The PLS-DA score plot demonstrates clear separation among the three clades (**Figure 6B**). This distinct spatial distribution strongly corroborates the classification patterns observed in the HCA result, validating the robustness of the chemical-based grouping.

To identify the key differential VOCs among the three clades, VIP analysis was used to evaluate the contribution of each VOC to clade differentiation (**Figure 6C**; **Supplementary Table 9**). The VIP plot represents the relative significance of several variables, with specific compounds, such as esters and hydrocarbons, exerting a more substantial influence on distinguishing the types across the three clades. A total of 75 VOCs with VIP scores >1.0 were identified, categorized as follows: 16 esters, 11 ketones, 11 hydrocarbons, 10 heterocyclic compounds,6 alcohols, 5 aldehydes, 5 acids, 1 terpenoids, 2 phenols, 2 ethers, 1 pyridine, and 5 other compounds (**Figure 6D**). Notably, seven VOCs exhibited particularly high discriminatory power, as indicated by their VIP scores greater than 1.7: acetic acid, (+)-cis-calamenene, 3-buten-1-ynylbenzene, propanoic acid, γ-acetopropanol, butyrolactone, and γ-terpinyl acetate. All seven compounds showed moderate relative abundance in Clade II. Among them, acetic acid, (+)-cis-calamenene, propanoic acid, and butyrolactone were most abundant in Clade I and lowest in Clade III; conversely, 3-buten-1-ynylbenzene, -acetopropanol, and γ-terpinyl acetate were highest in Clade III and lowest in Clade I. These pronounced abundance patterns across clades underscore their potential as chemical markers for differentiation.

### 3.4 Correlations among sensory attributes, biochemical, and VOCs

**Figure 7:**
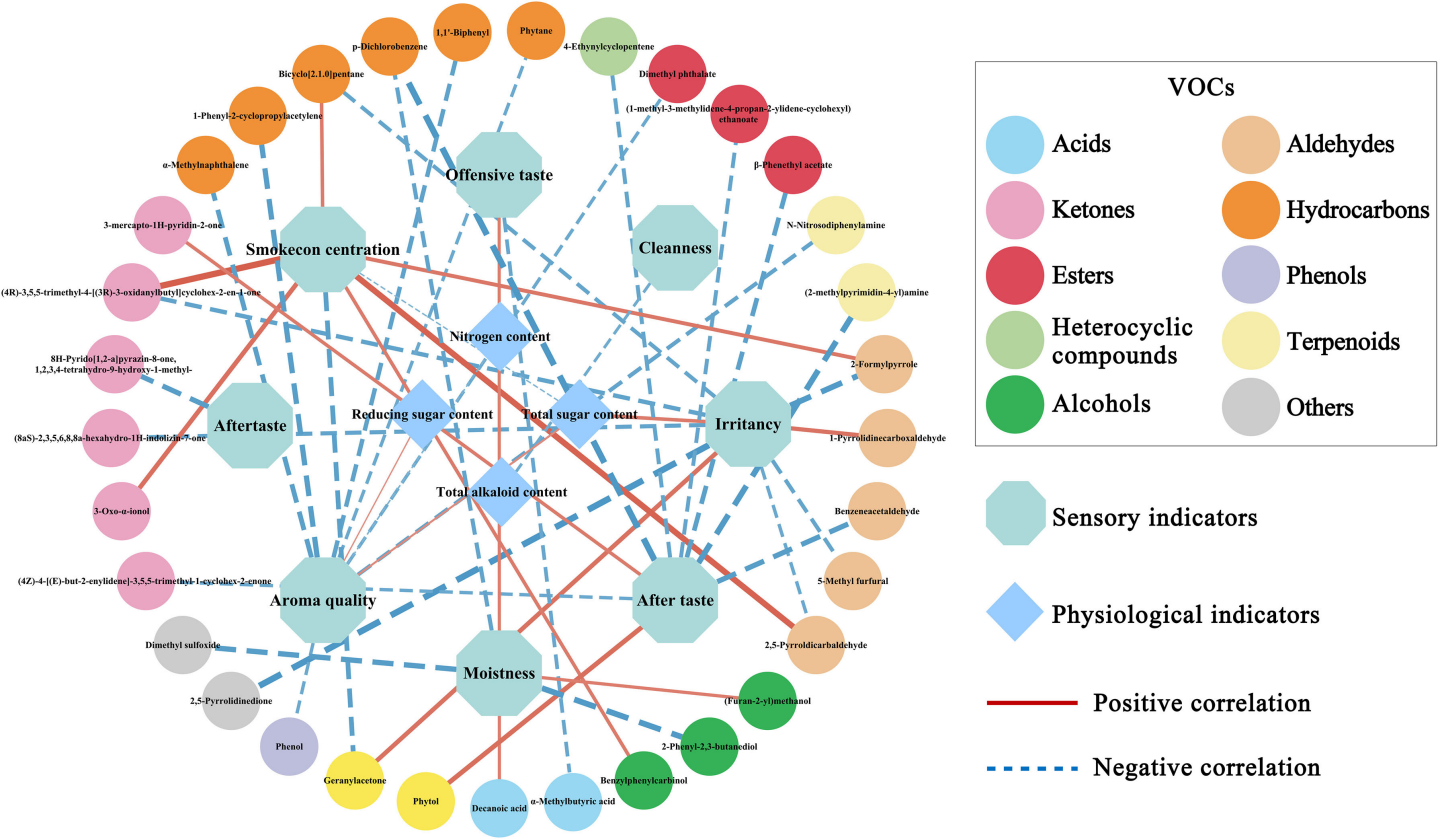
Network diagram showing significant associations between sensory evaluations, physiological indicators, and VOCs. Associations were determined using the Spearman method, with a significance threshold of *p* < 0.05 after Bonferroni correction. Line thickness represents the absolute value of the correlation coefficient.

To identify potential sensory markers, Spearman correlation analyses were conducted between sensory profiles and physiological indices, as well as between sensory profiles and VOCs. Significant associations were selected using a Bonferroni corrected *p*-value threshold of < 0.05. The results are presented in the **Figure 7**. Among the physiological indices, total sugar content showed significant correlations with irritancy (*r* = 0.70), aroma quality (*r* = 0.61), and smoke concentration (*r* = -0.57). Total alkaloid content was significantly negatively correlated with cleanness (*r* = -0.64). Reducing sugar content was positively correlated with aroma quality, whereas nitrogen content exhibited a negative correlation with aroma quality (**Supplementary Table 10**).

Additionally, 33 VOCs were significantly associated with six sensory attributes: Smokeconcentration, Irritancy, Aftertaste, Moistness, Aroma quality, and Offensive taste (**Supplementary Table 11**). The highest number of significant VOC correlations was observed for Aftertaste (10 VOCs), followed by Irritancy (9), Aroma quality (7), and Smoke concentration (7), whereas fewer VOCs were correlated with Moistness (4) and Offensive taste (2). The strongest absolute correlation coefficient was between Smoke Concentration and (4R)-3,5,5-trimethyl-4-[(3R)-3-oxidanylbutyl]cyclohex-2-en-1-one (*r* = 0.86), followed by that with 2,5-Pyrroldicarbaldehyde (*r* = 0.83). Notably, 12 of these 33 VOCs also had VIP scores greater than 1 in the PLS-DA analysis, underscoring their potential role in distinguishing sensory quality profiles.

## 4 Discussion

Cigar tobacco, derived from cured and fermented tobacco leaves, is a globally farmed economic crop known for its unique aroma and flavor. The environmental conditions and climate of tobacco cultivation regions predominantly influence the quality of cigarette tobacco leaves (Jia et al., 2022). Moreover, the fermentation method amplifies the flavor and aroma of tobacco leaves. This study employed a comprehensive, multi-faceted methodology—incorporating sensory evaluation, biochemical characterization, and VOC analysis—to analyze quality factors among eleven flue-cured tobacco cultivars cultivated in Yunnan. By examining pertinent research on analogous topics, including tobacco flavor assessment and the categorization of volatile chemicals, we can extract essential insights to elucidate the findings of this study.

The sensory panel characterized distinct sensory profiles among the eleven cultivars. No single cultivar was superior across all attributes; instead, each exhibited a unique combination of strengths. For instance, YN87 was distinguished by the highest aroma volume and the lowest irritancy, while XY7, YN105, GY2 and YN223 formed a top-tier group in aroma quality. YN228 presented a more balanced profile within the set. HCA and PCA both classified cultivars into two primary sensory groups, with YN228, YN105, and YN223 closely grouped (**Figure 2**). These patterns align with previous findings indicating that tobacco varieties group according to fragrance and irritancy as evaluated by expert panels (Krüsemann et al., 2019; Schwanz et al., 2019). For instance, similar to our PC1 (aroma volume/smoke concentration/irritancy) and PC2 (after taste/cleanness) axis, Krüsemann et al. (2019) demonstrated that defining cigarette smoke flavors (e.g., sweet, flowery, nutty) differentiates cultivars using sensory distance parameters. In a similar vein, Wang et al. (2016) employed sensory profiles to group flue-cured tobaccos into clusters based on flavor intensity, lending credence to the use of multi-axis sensory mapping for cultivar distinction. Significantly, our results enhance these findings by linking sensory clusters to biochemical and VOC profiles: Clade I (YN105, YN223) displayed higher scores in aroma quality, irritancy, and aftertaste, along with elevated levels of acids and total sugars as well as lower chloride content. This integrated clustering approach provides a more fundamental foundation than sensory data alone, confirming that “taste” and “smell” groupings arise from underlying chemical compositions (Jiang et al., 2024).

Our biochemical findings (**Figure 3**) support the well-documented influence of sugars, alkaloids, and mineral nutrients on tobacco sensory characteristics (Farrokh et al., 2012; Jiang et al., 2024). Significantly, cultivars YN223 and YN105 demonstrated the most prevalent total sugar content, corresponding with their exceptional aroma quality and mild irritancy. Sugars impart sweetness and serve as intermediates in Maillard reactions during curing, producing favorable caramel-like and nutty volatiles (Wahlberg et al., 1977). The positive associations between total sugar and aroma quality (*r* = 0.61) and irritancy (*r* = 0.70) (**Figure 7**) indicate that sugars enhance both sweetness and the sensory “kick” of smoke—a dual effect similarly reported by Seeman et al. (2003) in their study of sugar alcohols mitigating irritation.

Nicotine and other alkaloids constitute the basis of smoking potency and throat feeling (Andrade et al., 2018). Although YN87 and NC103 showed an high level in total alkaloids content, a significant inverse relationship was observed between total alkaloids and sensory cleanness (*r* = -0.64). This suggests that higher levels of alkaloids may negatively affect the perception of purity. This observation aligns with the findings of Farrokh et al. (2012), who noted that elevated nicotine (alkaloid) levels could contribute to a more severe experience. In flue-cured tobacco, alkaloids and other basic components interact with acidic constituents, directly influencing smoke attributes such as mildness, harshness, and cleanness (Davis and Nielsen, 1999). Chlorine and potassium govern combustion and can influence taste emission (Jia et al., 2022). Moreover, the elevated concentrations of chlorine in YN222, and nitrogen in NC103 and YN228, especially concerning their harmonious sensory profiles, underscore the significance of these elements in preserving aroma purity and mitigating harshness in tobacco smoke, consistent with prior findings (Jia et al., 2022). Meanwhile, balanced nitrogen levels improve fullness but may increase bitterness if excessive, as previously documented (Geng et al., 2025; Liu et al., 2022; Yoshida and Kobayashi, 2013). Collectively, our biochemical results demonstrate that an ideal sensory profile emerges from a meticulously calibrated interaction. Sufficient sugars to generate Maillard-derived fragrances, balanced alkaloids for desirable intensity, and minerals that regulate smoke delivery without harshness.

VOCs are essential in defining the olfactory character of tobacco, and their composition was meticulously examined in the present research. The findings indicated that esters (15.2%), ketones (14.7%), and hydrocarbons (13%) were the predominant VOC classes across all cultivars, aligning with Zhao et al. (2025), who noted that esters and hydrocarbons are essential in characterizing the aroma and aftertaste of different tobacco varieties. Additionally, 12 VOCs with VIP scores greater than 1 were recognized as discriminative markers among the VOC-based clades. These observed correlations can be reasonably interpreted based on compound origin and sensory properties. For example, 3-Oxo-α-ionol, a thermal oxidative degradation product of carotenoids, exhibited a significant positive correlation with smoke concentration. It is recognized as a key tracer of carotenoid pyrolysis in smoke (Wu et al., 2020). (Furan-2-yl)methanol, a marker of sugar and its thermal conversion products in tobacco, was positively correlated with moistness, a finding consistent with its reported contribution to smooth, sweet, and moist sensory notes. However, its reported associations with offensive taste and aftertaste in red sun-cured tobacco (Liu et al., 2016) differ from the present results, likely due to sample differences—the earlier study analyzed red sun-cured tobacco from eight Chinese regions, whereas this work focused on 11 flue-cured cultivars from a single region. Another study noted that (furan-2-yl)methanol had only a limited influence on the smoking quality of flue-cured tobacco (Cao et al., 2012). Thus, further investigation with expanded sample sets or directed manipulation of (furan-2-yl)methanol content is warranted to clarify its sensory impact. Phenol, a typical pyrolysis product of lignin and chlorogenic acid, showed a significant negative correlation with aroma quality. Phenol is described as “sharp,” “medicinal,” and irritating (Rodgman and Perfetti, 2013), and its elevated levels can disrupt aroma harmony by introducing undesirable off-notes, thereby lowering overall aroma quality scores.

Although this study integrated sensory, biochemical, and VOC data to identify several significant sensory-related components through correlation analysis, certain limitations should be acknowledged and addressed in future work. First, the findings are primarily based on 11 flue-cured cultivars from Yunnan province. Given the substantial chemotypic variation across cultivars and growing regions, subsequent studies should include a broader range of genotypes and geographical origins to identify sensory-relevant compounds with general applicability to flue-cured tobacco. Second, VOC quantification relied on peak-area normalization rather than calibration with authentic standards, which may influence correlation outcomes. Different normalization approaches—such as internal standard normalization versus total peak-area normalization—can yield markedly different association results (Noonan et al., 2018), as demonstrated in sensory-VOC studies of lily (Zhou et al., 2024) and *Hedychium* (Zhou et al., 2026) flowers. Therefore, future work should optimize VOC quantification methods, compare correlation patterns across different normalization strategies, and consider characterizing the sensory attributes or detection thresholds of key VOCs through techniques such as GC-olfactometry (GC-O).

## 5 Conclusions

This integrated analytical framework, combining sensory, biochemical, and VOC profiling, effectively characterized eleven flue-cured tobacco cultivars. Results revealed distinct, cultivar-specific profiles with no genotype superior across all sensory attributes. Multivariate analyses grouped cultivars based on shared chemical and sensory traits, while VIP analysis identified key discriminatory VOCs, including acetic acid and (+)-cis-calamenene. Biochemical components such as sugars and alkaloids correlated significantly with specific sensory attributes. The study demonstrates that targeted cultivar selection should prioritize trait combinations rather than overall superiority. This reproducible, multi-dimensional approach provides a robust foundation for quality-driven breeding and a structured framework for investigating genetic and environmental influences on tobacco quality.

## Data Availability

The original contributions presented in the study are included in the article/**Supplementary Material**. Further inquiries can be directed to the corresponding authors.
